# Conserved autophagy and diverse cell wall composition: unifying features of vascular tissues in evolutionarily distinct plants

**DOI:** 10.1093/aob/mcae015

**Published:** 2024-02-07

**Authors:** Kornel M Michalak, Natalia Wojciechowska, Katarzyna Marzec-Schmidt, Agnieszka Bagniewska-Zadworna

**Affiliations:** Department of General Botany, Institute of Experimental Biology, Faculty of Biology, Adam Mickiewicz University in Poznań, Uniwersytetu Poznańskiego 6, 61-614 Poznań, Poland; Department of General Botany, Institute of Experimental Biology, Faculty of Biology, Adam Mickiewicz University in Poznań, Uniwersytetu Poznańskiego 6, 61-614 Poznań, Poland; Nomad Foods Ltd, Findus Sverige AB, Bjuv, Sweden; Department of General Botany, Institute of Experimental Biology, Faculty of Biology, Adam Mickiewicz University in Poznań, Uniwersytetu Poznańskiego 6, 61-614 Poznań, Poland

**Keywords:** Evolution of vascular tissues, xylem, phloem, conductive cells, tracheary elements, sieve elements, autophagy, cell wall composition

## Abstract

**Background and Aims:**

The formation of multifunctional vascular tissues represents a significant advancement in plant evolution. Differentiation of conductive cells is specific, involving two main pathways, namely protoplast clearance and cell wall modification. In xylogenesis, autophagy is a crucial process for complete protoplast elimination in tracheary elements, whose cell wall also undergoes strong changes. Knowledge pertaining to living sieve elements, which lose most of their protoplast during phloemogenesis, remains limited. We hypothesized that autophagy plays a crucial role, not only in complete cytoplasmic clearance in xylem but also in partial degradation in phloem. Cell wall elaborations of mature sieve elements are not so extensive. These analyses performed on evolutionarily diverse model species potentially make it possible to understand phloemogenesis to an equal extent to xylogenesis.

**Methods:**

We investigated the distribution of ATG8 protein, which is an autophagy marker, and cell wall components in the roots of ferns, gymnosperms and angiosperms (monocots, dicot herbaceous plants and trees). Furthermore, we conducted a bioinformatic analysis of complete data on ATG8 isoforms for *Ceratopteris richardii*.

**Key Results:**

The presence of ATG8 protein was confirmed in both tracheary elements and sieve elements; however, the composition of cell wall components varied considerably among vascular tissues in the selected plants. Arabinogalactan proteins and β-1,4-galactan were detected in the roots of all studied species, suggesting their potential importance in phloem formation or function. In contrast, no evolutionary pattern was observed for xyloglucan, arabinan or homogalacturonan.

**Conclusions:**

Our findings indicate that the involvement of autophagy in plants is universal during the development of tracheary elements that are dead at maturity and sieve elements that remain alive. Given the conserved nature of autophagy and its function in protoplast degradation for uninterrupted flow, autophagy might have played a vital role in the development of increasingly complex biological organizations, including the formation of vascular tissues. However, different cell wall compositions of xylem and phloem in different species might indicate diverse functionality and potential for substance transport, which is crucial in plant evolution.

## INTRODUCTION

The transition in plant evolution from unicellular to multicellular organisms was associated with a selection pressure to evolve structures for intercellular exchange (Lucas *et al.*, 1993). This pressure intensified during terrestrialization and dramatic environmental changes (Kenrick and Crane, 1997; de Vries and Archibald, 2018). A subsequent fundamental evolutionary innovation involved the formation of cells capable of long-distance substance transport within more complex plant body organizations (reviewed by Lucas *et al.*, 2013). Despite the parallel evolution of bryophytes and vascular plants (Harrison, 2017), the development of conductive cells is associated with the reduction of cytoplasm content and modification of the cell wall for uninterrupted flow (Ligrone *et al.*, 2000; Ye, 2002; Pittermann, 2010; Heo *et al.*, 2017; Woudenberg *et al.*, 2022). However, the degree of conductive cell variation increased during evolution. The combination of all these adaptations in vascular plants, such as the presence of components enhancing transpiration and assimilation, in addition to an advanced conductive system, enabled them to attain large sizes and predominant colonization (Lucas *et al.*, 2013). Vascular bundles consist of heterogeneous tissues involved in developmental and physiological processes at the whole-plant level (Ye, 2002; Fukuda, 2004; Lucas *et al.*, 2013). Xylem and phloem are complex systems and, being constructed by several types of cells, have a common origin. Moreover, individual mature conductive elements are arranged end to end in tubular files and maintain a constant connection with the meristematic cells of their origin (Nakashima *et al.*, 2000; Scarpella *et al.*, 2004). Xylem comprises tracheary elements (TEs) responsible for water and mineral transport, parenchyma protecting against cavitation and embolism, and fibres providing mechanical support (Bagniewska-Zadworna *et al.*, 2014; Venturas *et al.*, 2017; Słupianek *et al.*, 2019, 2021). Phloem includes sieve elements (SEs) responsible for sugar allocation and transport of various signalling molecules, companion cells keeping SEs alive, parenchyma participating in sugar loading, and fibres exerting mechanical functions (Ruiz-Medrano *et al.*, 2001; van Bel *et al.*, 2002; van Bel, 2003; Evert, 2006; Lough and Lucas, 2006). Interestingly, developmental programmes have evolved such that mature TEs are dead and SEs are living cells but have significantly reduced cytoplasm (Ye, 2002; Fukuda, 2004; Lucas *et al.*, 2013). Thus, complete and partial degradation mechanisms are involved in xylogenesis and phloemogenesis, respectively (Wojciechowska *et al.*, 2021). TEs lose protoplast during differentiation through programmed cell death (PCD) (Kwon *et al.*, 2010*b*; Bagniewska-Zadworna *et al.*, 2012; Escamez *et al.*, 2016), and similar processes occur in developing SEs (Wang *et al.*, 2008; Furuta *et al.*, 2014; Yang *et al.*, 2015). One common pathway for cytoplasmic removal is autophagy, which has been documented in the development of both TEs and SEs (Wojciechowska *et al.*, 2019). Another essential process in the differentiation of conductive cells is cell wall remodelling (Demura and Fukuda, 1994). Polysaccharides are core elements that vary the wall composition according to the function of the cell (Mellerowicz and Sundberg, 2008; Caffall and Mohnen, 2009; Marzec-Schmidt *et al.*, 2019). Furthermore, genetic studies have revealed the evolutionary conservation of molecular networks regulating both protoplast degradation and cell wall modification among vascular plants (Ohtani *et al.*, 2017).

**Fig. 1.**
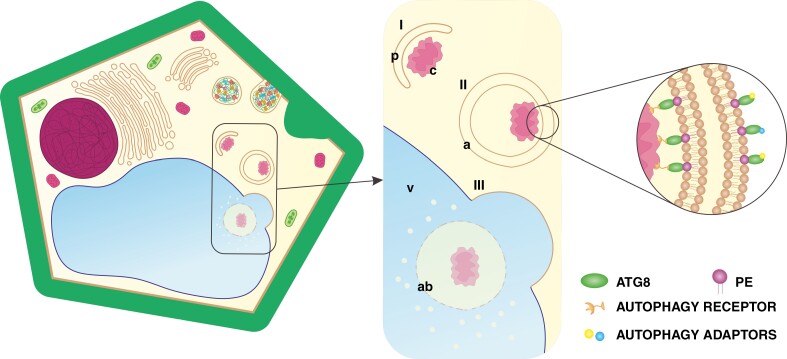
Schematic representation of autophagy in plant cells. The process consists of: (I) phagophore (p) incorporating cargo (c); (II) autophagosome (a) maturation; and (III) autophagosome fusion with the vacuole (v), followed by the release of the autophagic body (ab) for digestion. Abbreviation: PE, phosphatidylethanolamine. (Not drawn to scale.)

Macroautophagy (hereafter autophagy) is a tightly regulated degradation pathway for cytoplasmic materials, macromolecules or organelles. It facilitates nutrient remobilization, maintenance of homeostasis or removal of protoplast elements (Li and Vierstra, 2012; Ding *et al.*, 2018; Klionsky *et al.*, 2021). Initially, autophagy forms a phagophore, which sequesters cell components for degradation (Fig. 1I). The phagophore then develops into a double-membrane vesicle called an autophagosome (Fig. 1II) that traffics cargo to the vacuole for digestion (Fig. 1III) (Avila-Ospina *et al.*, 2014; Yu and Melia, 2017; Soto-Burgos *et al.*, 2018). Autophagy is highly conserved, because most of the ~40 autophagy-related (ATG) proteins are encoded by orthologues with high sequence similarity in eukaryotes. Consequently, the pathway of autophagic events is universal for all organisms. ATG8 plays a dominant role, because it is the only protein indispensable at every stage of autophagy (Klionsky and Schulman, 2014; Kellner *et al.*, 2017; Bu *et al.*, 2020). Complexed with phosphatidylethanolamine (PE), ATG8 is inserted into the membranes of autophagy structures (Klionsky and Schulman, 2014; Thukral *et al.*, 2015; Maruyama *et al.*, 2021). ATG8 in the inner membrane binds to specific autophagy receptors (Fig. 1), forming an autophagy interacting motif responsible for cargo recruitment (Honig *et al.*, 2012; Kellner *et al.*, 2017; Nolan *et al.*, 2017; Gatica *et al.*, 2018; Marshall *et al.*, 2022). ATG8 in the outer membrane associates with autophagy adaptors (Fig. 1) and participates in delivery of autophagosomes to the vacuole and in fusion with the tonoplast (Ketelaar *et al.*, 2004; Yoshimoto *et al.*, 2004; Yu and Melia, 2017; Brillada *et al.*, 2018). Considering this evidence supporting the dominant role of ATG8 in autophagy, the protein has been established as a molecular marker for this process. The investigation of its diversity across 58 plant species has revealed different numbers of isoforms per taxon (Kellner *et al.*, 2017), but functional differences between them are unknown. Autophagy participates in PCD, but depending on the stimuli, it can be activated to obtain necessities from alternative sources or to remove only elements precisely selected by autophagy interacting motifs (Minina *et al.*, 2014). Thus, the evolution of the ATG8 protein might have influenced the development of conductive systems in plants.

**Fig. 2.**
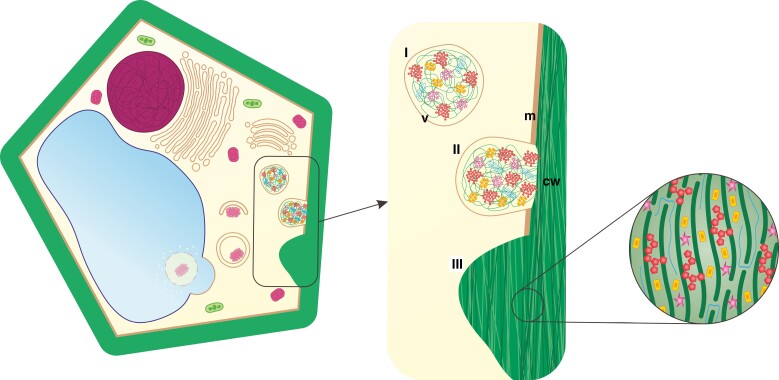
Schematic representation of plant cell wall formation: (I) formation of a vesicle (v) with cell wall matrix; (II) compounds modifying cell wall (cw) chemistry are delivered via exocytosis (m, cell membrane); and (III) the secondary cell wall is highly differentiated and can form thickenings. (Not drawn to scale.)

In addition to autophagy, cell wall modification appears crucial for the effective functioning of conductive elements through uninterrupted flow. It provides cell wall protection against physical factors, determines their shape and prevents them from collapsing into the cell. The cell wall is composed of various substances, and its composition depends on the function performed in the body of the plant. A common feature is the presence of biopolymers (mainly cellulose) forming its network. Moreover, chemical complexity determines whether the cell wall acts as a barrier or a transport site (Sakurai, 1991; Burton *et al.*, 2010). During tissue differentiation, various components are incorporated into the cell wall (Fig. 2) by components delivered by vesicles with cell wall matrix (Fig. 2I), providing the structure with both relative rigidity and a high degree of flexibility. The thickness of the cell wall changes by modifying the primary cell wall or by forming the secondary cell wall. Components that shift the structure and function are secreted precisely into the cell wall during development (Fig. 2II; Fry, 2004; Zhang *et al.*, 2021; Cosgrove, 2022). In plants, the main polysaccharide with photosynthetically fixed carbon is cellulose (Popper, 2008), but polysaccharide composition can differ dramatically depending on the tissue, the organ, the step of development or the species, including cell wall components such as pectin homogalacturonan (HG), non-cellulosic β-1,4-linked glucan, hemicellulose xyloglucan, arabinan and arabinogalactan (AG), often connected with proteins (AGP) (Caffall and Mohnen, 2009; Schneider *et al.*, 2016; Hofte and Voxeur, 2017). However, lignin is the most abundant biopolymer after cellulose, specific to some secondary cell walls (Whetten *et al.*, 1998; Boerjan *et al.*, 2003), which starts to alter cell wall properties after cell expansion has ceased and forms thickenings (Fig. 2III), even post-mortem in the case of TEs (Pesquet *et al.*, 2013; Smith *et al.*, 2013; Mishima *et al.*, 2014). Collectively, developed strategies of cell wall construction have established the structural and functional diversity of tissues (Sorensen *et al.*, 2010; Fangel *et al.*, 2012; Niklas *et al.*, 2017; Little *et al.*, 2018). The programmed deposition of patterned material within the cell wall was an evolutionary event that imparted biomechanical support and the ability for long-distance transport of substances (Popper *et al.*, 2011; Banasiak, 2014). However, the level of similarity in cell wall composition of vascular tissues is unknown for plant species from different lineages.

The spatiotemporal molecular framework of procambium differentiation into vascular precursors and subsequent maturation of TEs and SEs remains elusive (Fukuda, 2004; Lucas *et al.*, 2013). However, molecular regulators of PCD and cell wall modification are fundamental during the development of vascular tissues (Ohtani *et al.*, 2017). Autophagic clearance of protoplasts (Wojciechowska *et al.*, 2019, 2021) and cell wall remodelling (Popper *et al.*, 2011) are evolutionary innovations thought to contribute to developmental dynamics and the specification of structures for effective long-distance substance transport in vascular plants. However, data on these processes have been referred to for only a few model organisms and are predominantly focused on xylem. Two reasons contribute to a better understanding of vascular tissue development: (1) the recognition of autophagy function in both TEs and SEs; and (2) the diversity of cell wall components in these conductive cells. A fundamental question in plant functional anatomy is to determine whether autophagy is a characteristic during the development of TEs, which are dead at maturity, and whether it also occurs during differentiation of SEs, which remain alive. Furthermore, it is important to compare the composition of cell walls of vascular tissues considering evolutionary adaptation. Considering autophagy and cell wall modification as two main processes responsible for the development of vascular tissues in plants, this study contributes to identification of hypothetical evolutionary patterns that can influence their functionality explained by the life history of the studied species. To tackle these challenges, we present the localization of the autophagy marker ATG8 along with selected cell wall-related components in xylem and phloem of evolutionarily diverse vascular plants.

## MATERIALS AND METHODS

### Plant material

Vascular plant species of model organisms from diverse lineages (fern, gymnosperm and angiosperm: monocotyledonous and dicotyledonous, both herbaceous and woody) were selected for the experiments. The fern *Ceratopteris richardii* was cultivated from spores obtained from the Carolina Biological Supply Company (Burlington, NC, USA). The gymnosperm *Picea sitchensis*, the monocotyledon *Zea mays* and the herbaceous dicotyledonous *Arabidopsis thaliana* were acquired from seed and horticultural companies. Samples of the dicotyledonous tree *Populus trichocarpa* were collected at an experimental field site, as previously described (Wojciechowska *et al.*, 2018). All experiments were conducted on roots in the differentiating state specific to each species (Supplementary Data Table S1). At least five root fragments of different plants from each species were subjected to studies.

### Gene identification

The number of sequences encoding ATG8 proteins for the selected plant species was determined based on a prior phylogenetic overview (Kellner *et al.*, 2017). For *C. richardii*, a genomic sequence (Marchant *et al.*, 2019, 2021; Geng *et al.*, 2021) was screened for ATG8 homologues using the BLASTP tool. The ATG8a protein of *A. thaliana* was chosen as a query for sequence alignment. Potential sequences that overlapped with the AtATG8a protein were found and, according to similarity searches (Kellner *et al.*, 2017), only sequences that contain the C-terminal glycine and cover all ATG8-specific α-helices, β-sheets and the conserved ATG8 domain (P02991) were selected (Supplementary Data Table S2). Transcripts of the same gene and repetitive sequences were removed from further analysis.

### Multiple sequence alignment and phylogenetic analysis

Multiple sequence alignments of a total of 34 selected ATG8 proteins from selected plant species were completed using ClustalW alignment in MEGA 7.0 (Kumar *et al*, 2016) (Supplementary Data Fig. S1). A phylogenetic tree (Supplementary Data Fig. S2) was constructed using MEGA 7.0 with neighbour-joining and 1000 replicates of the bootstrap test and pairwise deletion.

### Anatomical studies

Harvested root fragments were immediately fixed in a mixture of 2 % (v/v) glutaraldehyde (pH 6.8; Polysciences, Warrington, PA, USA) and 2 % (v/v) formaldehyde (pH 6.8; Polysciences). After overnight incubation in the fixative solution, samples were rinsed three times with a cacodylate buffer (0.05 m; pH 6.8; Polysciences) and subsequently dehydrated in a graded ethanol series (10–100 %, v/v). The samples were then incubated in a series of ethanol: Technovit 7100 resin mixtures (Heraeus Kulzer, Wehrheim, Germany) with ratios of 3:1, 1:1 and 1:3, and finally in pure Technovit 7100 resin. Cross-sections were obtained using a Leica RM2265 Fully Automated Rotary microtome (Leica-Reichert, Bensheim, Germany) at a thickness of 10 μm. These cross-sections were stained with 0.5 % (m/v) Toluidine Blue (pH 6.8) and examined under a light microscope (Axioscope A1, Carl Zeiss, Jena, Germany).

### Immunolocalization of ATG8 and cell wall components

For ATG8 detection, root fragments were cut into 40-μm-thick slices using a Leica VT 1200S (Leica Biosystems, Nussloch, Germany) vibratome. Sections were fixed in 4 % (v/v) formaldehyde (pH 6.8; Polysciences) and 0.5 % (v/v) glutaraldehyde (pH 6.8; Polysciences) for 2 h, then rinsed in 0.01 m phosphate-buffered saline (PBS; Sigma-Aldrich, USA) buffer. For membrane permeabilization, the material was incubated for 15 min in 0.1 % Triton (Sigma-Aldrich, USA) followed by incubation for 1 h at room temperature in 3 % hydrogen peroxide solution to quench endogenous peroxidase activity. Subsequently, the material was rinsed and treated with antibodies according to the procedure described by Wojciechowska *et al.* (2019). The tyramide signal amplification (TSA) technique was used because of its high level of sensitivity, as reported by Wojciechowska *et al.* (2018). A primary ATG8 rabbit antibody (catalogue no. AS14 2769; Agrisera, Sweden) was diluted 1:1000 with 0.2 % bovine serum albumin (BSA)/PBS and used in conjunction with a poly-horseradish peroxidase-conjugated secondary antibody (Thermo Fisher Scientific Inc., USA, attached to the TSA Super Boost kit).

For detection of cell wall components, roots were immediately fixed in a mixture of 2 % (v/v) glutaraldehyde (pH 6.8; Polysciences) and 2 % (v/v) formaldehyde (pH 6.8; Polysciences) and embedded in Paraplast X-TRA^®^ (Sigma-Aldrich, USA). Cross-sections were cut using a Leica RM2265 Fully Automated Rotary microtome (Leica-Reichert) at a thickness of 15 μm. Different IgG rat primary antibodies were applied according to the procedure described by Marzec-Schmidt *et al.* (2019). Monoclonal antibodies (Supplementary Data Table S3) were diluted 1:10 with 0.2 % BSA/PBS. Secondary antibody goat anti-rat IgG (H + L), Alexa Fluor^®^ 488 Conjugate (catalogue no. 31470; Thermo Fisher Scientific Inc., USA) was diluted 1:100 with 0.2 % BSA/PBS. For arabinogalactan protein (AGP) LM2 or LM14 antibodies (Plant Probes) and for HG LM7, LM18, LM19 or LM20 antibodies (Plant Probes) were tested on roots of *Populus trichocarpa* to check similarity in signal distribution within the vascular cylinder. In order not to duplicate the results for the same component, LM2 and LM18 were chosen owing to high specificity for vascular tissues (Supplementary Data Fig. S3). For β-1,4-galactan LM5, for xyloglucan LM15 and for arabinan LM16 antibodies (Plant Probes) were used. The material was mounted in Prolong Gold (Life Technologies, Thermo Fisher Scientific Inc., USA).

Results of the immunolocalization assay were recorded using a Leica TCS SP5 confocal microscope (Leica Biosystems) with a 405 nm diode laser for observing autofluorescence of lignins and an argon laser emitting light at 488 nm for observing Alexa 488 fluorochrome fluorescence. At least five root segments from each species were analysed for each tested antibody. Incubations without primary antibodies served as negative controls. No detectable results were obtained with the negative controls.

## RESULTS

Bioinformatic analyses have shown that the number of sequences encoding (cdsSeq) ATG8 proteins increases in more recently evolved plants. In *C. richardii*, a representative of ferns, six sequences that overlapped with *AtATG8a* have been identified. However, only three of them encode proteins containing the C-terminal glycine and cover all ATG8-specific α-helices and β-sheets. This is more than for *Picea sitchensis*; however, in *Populus trichocarpa*, which has the most complex vasculature, there are as many as 14 of them (Supplementary Data Table S2). To evaluate the evolutionary relationship among *ATG8* genes of plants, an unrooted neighbour-joining tree was constructed derived from *C. richardii*, *Picea sitchensis*, *Z. mays*, *A. thaliana* and *Populus trichocarpa* ATG8 protein sequences. The proteins were divided into six clades (Supplementary Data Fig. S2). We identified that one of the fern ATG8 proteins (Ceric.21G035500.1) grouped separately from the other species and that two others (Ceric.27G065300 and Ceric.33G046800) clustered within a clade containing, among others, AtATG8e, AtATG8f and AtATG8g. Interestingly, also one of the spruce ATG8 proteins grouped separately from the others (EF084104.1). All maize ATG8 proteins clustered together as a separate group. Poplar ATG8 family members were clustered with their *Arabidopsis* protein orthologues, which indicates that poplar ATG8 genes were phylogenetically more closely related to those of *Arabidopsis* than to those of fern and maize. The accession numbers and nucleotide sequences of all studied species are provided in the Supplementary Data (Table S2).

**Fig. 3.**
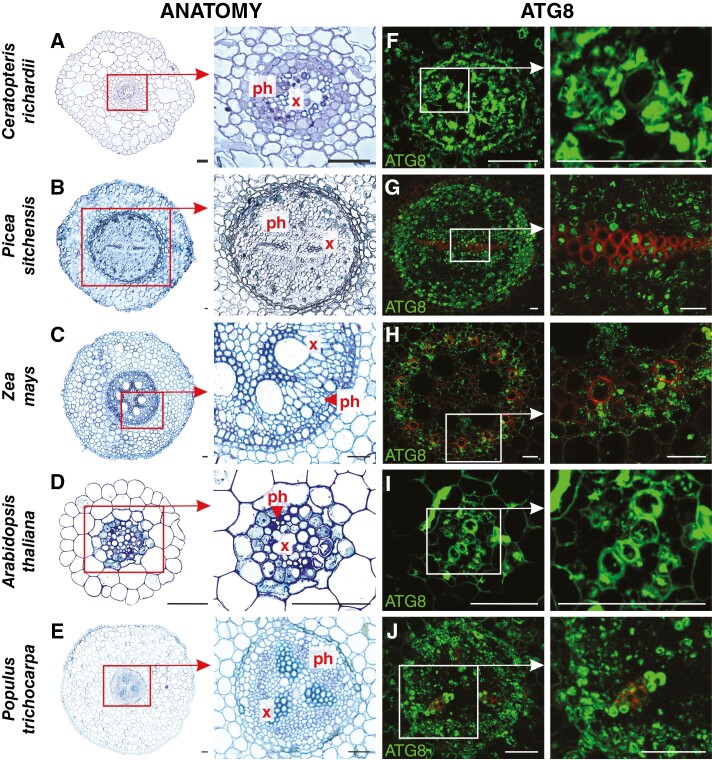
Anatomy (A–E) and ATG8 immunolocalization (green fluorescence; F–J) in selected vascular plant model organisms. Abbreviations: ph, phloem; x, xylem. Scale bars: 50 μm.

An anatomical analysis was conducted to identify components of vascular tissue and to recognize the ATG8 protein distribution inside. ATG8 epitopes were recognized in the area of vascular tissues in every species analysed; however, their distribution and the intensity of the fluorescent signal were heterogeneous. Results for each species are presented chronologically according to the course of evolution. In *C. richardii* roots, a single small vascular cylinder is encircled by endodermis in the middle. The central strand of xylem is surrounded by phloem and cortical parenchyma (Fig. 3A). A fluorescent signal indicating ATG8 localization was observed in both tracheids and phloem elements (Fig. 3F). In *Picea sitchensis*, the vascular system is more complex and occupies a larger part of the root. A radial cylinder is constructed by two strands of xylem arranged in the shape of an ellipse, with phloem strands on both sides (Fig. 3B). ATG8 was localized in conductive cells of xylem and phloem. There was a weaker signal in parenchyma and endodermis; however, between these tissues, a signal was observed in the multi-layered proliferating pericycle (Fig. 3G). In the roots of *Z. mays*, the vascular cylinder has an alternating arrangement of multiple xylem and phloem poles separated by parenchymatous cells. Metaxylem vessels with large diameters are distinguishable from much narrower protoxylem vessels. The phloem strands are adjacent to pericycle cells (Fig. 3C). A signal was dispersed in the vascular bundle, the strongest in protoxylem and phloem cells (Fig. 3H). The vascular cylinder in the primary structure of *A. thaliana* root is diarchic, with xylem strands connecting in the middle and phloem poles alternating on both sides. Vascular tissues are surrounded by one layer of pericycle and subsequently by endodermis (Fig. 3D). ATG8 was localized in developing TEs and phloem. Additionally, the signal was observed in single cells of endodermis (Fig. 3I). In *Populus trichocarpa*, the primary structure stele is arranged in a triarchic or tetrarchic organization. Large strands of xylem and phloem are constructed by multiple conductive cells (Fig. 3E), in which a fluorescent signal was documented. Xylem and phloem poles are separated by parenchyma with no signal; however, ATG8 was also localized in parenchyma between the phloem and pericycle (Fig. 3J). Negative control reactions revealed an undetectable signal compared with the standard reactions (Supplementary Data Fig. S4).

**Fig. 4.**
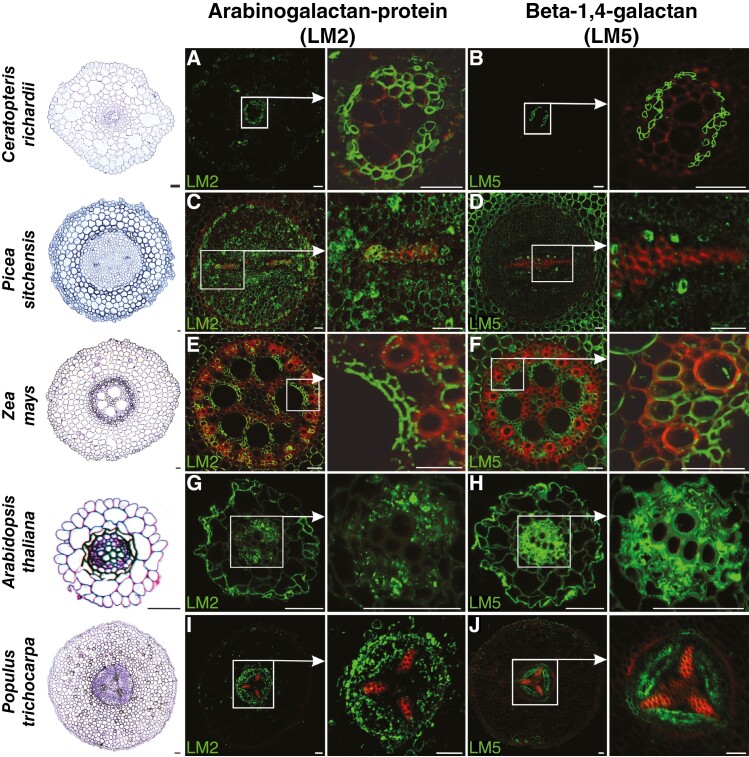
Immunolocalization of arabinogalactan protein (LM2) and β-1,4-galactan (LM5) in roots of plants belonging to various taxonomic groups (fern, gymnosperm and angiosperm: monocotyledonous and dicotyledonous, both herbaceous and woody). Red colour indicates lignin autofluorescence. Scale bars: 50 μm.

**Fig. 5.**
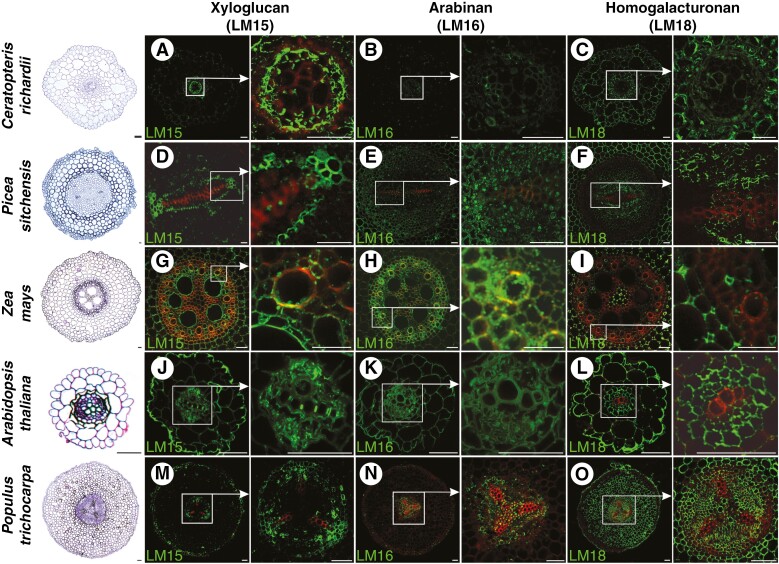
Immunolocalization of xyloglucan (LM15), arabinan (LM16) and homogalacturonan (LM18) in roots of plants belonging to various taxonomic groups (fern, gymnosperm and angiosperm: monocotyledonous and dicotyledonous, both herbaceous and woody). Red colour indicates lignin autofluorescence. Scale bars: 50 μm.

Apart from the complete and partial degradation of cellular components in conductive cells of xylem and phloem, respectively, another significant process related to development of vascular tissue is the remodelling and/or formation of the secondary cell wall. To determine the differences in cell wall composition in the primary xylem and phloem of various plant species belonging to different evolutionary lineages, immunolocalization reactions were conducted (Figs 4 and 5) using monoclonal antibodies against AGP (LM2), β-1,4-galactan (LM5), xyloglucan (LM15), arabinan (LM16) and HG (LM18).

For AGP localization, the LM2 antibody, recognizing a carbohydrate epitope containing β-linked glucuronic acid, was used (Smallwood *et al.*, 1996; Yates *et al.*, 1996). AGPs were highly localized in phloem cells, and such a result with an intense signal was confirmed for all studied species (Fig. 4A, C, E, G, I). However, in xylem, the distribution pattern of AGPs was not as comprehensive (Table 1). In ferns, gymnosperms and woody angiosperm plants, AGPs were evident, especially in protoxylem (Fig. 4A, C, I). In monocots, the intensive signal of immunostaining reaction was observed mainly in parenchyma xylem cells encircling metaxylem vessels (Fig. 4E). No AGP labelling was observed in xylem for herbaceous plants (Fig. 4G). AGP localization using LM14 antibody recognizing the glucuronosyl residue of AGP differed slightly in the root of *Populus trichocarpa*. Fluorescent signal was stronger and less specific in the area of vascular tissues (Supplementary Data Fig. S3A, B).

**Table 1.** Specific distribution of cell wall components in phloem and xylem in roots of plants belonging to various evolutionary lineages (fern, gymnosperm and angiosperm: monocotyledonous and dicotyledonous, both herbaceous and woody).

Component	*Ceratopteris richardii*	*Picea sitchensis*	*Zea* *mays*	*Arabidopsis thaliana*	*Populus trichocarpa*
**PHLOEM**					
Arabinogalactan protein (LM2)	+++	+++	++	+++	+++
β-1,4-Galactan (LM5)	+++	+	++	+++	+++
Xyloglucan (LM15)	+++	−	−	++	+
Arabinan (LM16)	−	+/−	++	−	+/−
Homogalacturonan (LM18)	−	++	+	++	+++
**XYLEM**					
Arabinogalactan protein (LM2)	+++^d^	++^d^	++^c^	−	+^d^
β-1,4-Galactan (LM5)	−	+/−	++^c^	+	−
Xyloglucan (LM15)	+++^b^	+/−	++	++^b^	+++^b^
Arabinan (LM16)	−	+/−	++	+/−	++^a^
Homogalacturonan (LM18)	**−**	**−**	**−**	**−**	**−**

Symbols: +++ or ++, binding; +, weak binding; +/−, negligible binding; −, lack of binding.

^a^Signal was localized only in the immature xylem cells.

^b^Signal was localized especially in the secondary cell wall thickening.

^c^Signal was localized especially in the parenchyma cells surrounding the metaxylem.

^d^Signal was localized especially in the protoxylem.

Galactan localization was performed using LM5 antibodies recognizing a linear tetrasaccharide in (1-4)-β-d-galactans (Jones *et al.*, 1997). For almost all studied species (Fig. 4B, F, H, J), including several cells in *Picea sitchensis* (Fig. 4D), the immunolabelling reaction confirmed the localization of galactan in phloem cells. In xylem, the presence of galactan was confirmed in conductive elements only for *A. thaliana* (Fig. 4H) and *Z. mays*, and in the latter species the signal was detected mainly in xylem parenchyma cells (Fig. 4F).

For xyloglucan localization, LM15 antibodies recognizing the XXXG motif were employed (Marcus *et al.*, 2008). However, a consistent pattern of signal distribution was not observed among the studied species in both phloem and xylem tissues (Table 1). Xyloglucan was predominantly observed in the cell walls of phloem in two species: *C. richardii* and *A. thaliana* (Fig. 5A, J). A faint signal was also detected in *Populus trichocarpa* phloem cells (Fig. 5M). In xylem cell walls, the presence of xyloglucan was confirmed for *C. richardii* (Fig. 5A), *Z. mays* (Fig. 5G) and *A. thaliana* (Fig. 5J). In *C. richardii* and *A. thaliana*, the signal appeared in cell walls with secondary cell wall thickenings. Furthermore, positive immunolabelling results were identified in procambial/cambial cells of species capable of developing secondary growth, such as *Picea sitchensis*, *A. thaliana* and *Populus trichocarpa* (Fig. 5D, J, M).

For arabinan distribution, monoclonal antibodies LM16, which recognize a processed arabinan/RG-I epitope associated with arabinans, were used (Verhertbruggen *et al.*, 2009). The majority of the studied species exhibited weak (Fig. 5E, K) or even no signal (Fig. 5B) of arabinan immunolabelling in vascular tissues (Table 1). A relatively intense signal was detected solely in xylem and phloem cells of *Z. mays* and *Populus trichocarpa* (Fig. 5H, N).

Homogalacturonan deposition was analysed using LM18 antibodies, which preferentially bind to partly methylesterified HG epitopes and un-esterified HG (Verhertbruggen *et al.*, 2009). HG labelling was abundantly observed in phloem cell walls of all studied species (Fig. 5F, I, L, O), except *C. richardii* (Fig. 5C). No labelling or barely visible HG labelling was documented for xylem cells. A highly intense signal was detected in parenchyma cortex cells across all studied species. Signals identifying HG localization in the root of *Populus trichocarpa* using other antibodies were similar. For LM7, recognizing partly methyl esterified HG, the signal in phloem and parenchyma of vascular tissues was observed. The same signal was observed for LM20 recognizing methyl esterified HG; however, it also binds to parenchyma cortex cells. Weaker signal was detected in the area of vascular tissues by LM19 antibody binding to unesterified HG (Supplementary Data Fig. S3C**–**F).

## DISCUSSION

The formation of TEs and SEs represented a crucial advancement in plant evolution, because conducting cells effectively addressed the challenges of continuous long-distance transport, provided mechanical support, and influenced plant productivity and environmental adaptation. TEs, as water- and nutrient-conducting elements, are dead and formed during xylogenesis, a multi-stage process, in which the final step involves bulk degradation of the protoplast, governed by mechanisms of developmental PCD and secondary cell wall thickenings (Turner *et al.*, 2007). The precise removal of all cytoplasmic elements is facilitated by autophagy (Li and Vierstra, 2012; Ding *et al.*, 2018). We highlighted attributes that could be explained by life history, thus our results documented the activation of this mechanism in TEs among species across diverse evolutionary lineages. We focused on the ATG8 protein, essential in all steps of autophagy flux and a molecular marker of this process (Klionsky and Schulman, 2014; Kellner *et al.*, 2017; Klionsky *et al.*, 2021), indicating its occurrence in differentiating TEs in all organisms studied. It was described in detail at the ultrastructural level (Kwon *et al.*, 2010*a*; Bagniewska-Zadworna *et al.*, 2012) and the molecular level (Wojciechowska *et al.*, 2019). It appears that, unlike autophagy, there is no conserved evolutionary pattern in cell wall composition of TEs. However, both analysed traits seem to be crucial for such extensive transport of substances through xylem. Consequently, our present results, combined with previously obtained data, distinctly indicate that only autophagic clearance of the protoplast, enabling the production of functional TEs, is a universal process for vascular plants. Surprisingly, our evaluation of published studies revealed a lack of detailed data on SEs. We proposed that autophagy could also be a crucial and essential process involved in phloemogenesis. Although SEs are living cells, they lack a nucleus and have a cytoplasm devoid of organellar content. Autophagy underlies the bulk degradation of protoplasts in TEs; however, selective autophagy might be responsible for the depletion of cytoplasmic content in SEs (Wojciechowska *et al.*, 2021). The presence of ATG8 during xylem and phloem development in various vascular plants suggests that autophagy is a universal and widespread process in the differentiation of both TEs and SEs. Moreover, we demonstrated that autophagy already occurred during the development of vascular tissues in pteridophytes.

Extensive phylogenetic analyses have confirmed that ATG8 sequences have remained relatively unchanged across eukaryotes throughout evolution. Similar or even identical binding molecules can be used for its detection in species from other kingdoms. Given the conservation and the principal role of ATG8 in autophagy, this protein is the most feasible tool for monitoring autophagy. In contrast to algae, which possess only one ATG8 isoform, for flowering plants both expansion and extinction have been involved in the evolution of the ATG8 gene family (Kellner *et al.*, 2017). Our analyses revealed four sequences encoding ATG8 for the fern *C. richardii*, which is more than for the gymnosperm *Picea sitchensis* (three isoforms); however, in general, the number of ATG8 isoforms increases throughout plant evolution. This dominant expansion has contributed to specific sets of ATG8 isoforms for each plant family. Intriguingly, it might have been related to interactions with individual autophagy interacting motif-containing proteins, i.e. ATG8 functionality (Kellner *et al.*, 2017), but this remains unclear. Undoubtedly, ATG8 proteins have been regarded as a central player in cargo recognition during autophagy. Owing to their unique amino acid sequences, different isoforms bind to specific receptors, creating different motifs. These, along with the interaction of other autophagy-related proteins, determine selective autophagy pathways (Luo *et al.*, 2021). However, ATG8 is also a driver involved in PCD processes. Owing to the involvement of autophagy in xylogenesis, which ends with cell death, and phloemogenesis, with selective removal of cytoplasm in vascular plants, it is imperative to fill gaps in our knowledge of individual ATG8 isoform specificity for the morphodynamics of TEs or SEs. ATG8 isoform diversity might have an impact on bulk or partial degradation pathways.

Autophagy is also responsible for regulating homeostasis and developmental processes (Klionsky *et al.*, 2021). It is suggested that bulk or selective degradation of protoplasts involved in tissue development are later functions of autophagy, specific to higher plants. Various *ATG8s* are up- or downregulated in vascular tissues. In *Populus trichocarpa*, expression of *ATG8c* primarily increases with progressive development of vascular tissue in pioneer roots, whereas *ATG8h* expression increases in stems (Wojciechowska *et al.*, 2019). These findings indicate an intriguing possibility that different ATG8 isoforms might have variable specificity and can be assigned functionally to respond to various factors at each stage of tissue or organ development. Moreover, the expression pattern of each *ATG8* varies among organs and through growth stages in plants of different species (Yoshimoto *et al.*, 2004; Thompson *et al.*, 2005; Shibuya *et al.*, 2011; Sanchez-Vera *et al.*, 2017). Given that ATG8 is involved in developmental processes and is abundant in differentiating both TEs and SEs in plants from different lineages, the evolution of autophagy, determined by the expansion of ATG8 isoforms, might have influenced the range of autophagy roles, resulting in the development of advanced conductive systems. Moreover, ATG8 was localized in parenchyma cells. In fact, autophagy is not always related to PCD. This process is responsible for vacuole biogenesis and the formation of the dominant central vacuole (van Doorn *et al.*, 2015; Machado and Rodrigues, 2019), in addition to maintenance of homeostasis (Inoue *et al.*, 2006; Yano *et al.*, 2007). It is also activated during dynamically dividing cells (Cheng at al., 2022), given that we showed ATG8 occurrence in the pericycle. It is possible that ATG8 found in endodermis is related to an autophagy role in starch biosynthesis (Sakil *et al.*, 2022). However, there is a lack of knowledge on the function of autophagy in the development of each tissue category.

Considering the highly conservative nature of autophagy, the increasing number of ATG8 isoforms and the evolutionary adaptation of plants, it is possible that autophagy has enhanced and contributed to the advancement of multi-component tissues. In conjunction with the proven involvement of autophagy in vascular tissue development, an intriguing possibility is that the evolution of autophagy might affect the formation of more complex conducting systems capable of efficient substance transport.

Another crucial process in the development of conducting cells is the formation and modification of cell walls. Cell wall remodelling and autophagy operate simultaneously during the differentiation of vascular tissues. Autophagic events and Golgi apparatus-derived vesicles, containing matrix substances inserted into the cell wall, were observed during xylogenesis in the roots of *Populus trichocarpa* (Bagniewska-Zadworna *et al.*, 2012). These processes provided heterogeneity in the vascular bundle, exquisitely suited to the requirements that arose during evolution (Popper *et al.*, 2011). Our studies identified distinct distribution patterns of cell wall components in functioning conductive cells between TEs and SEs and among various vascular plants. Cell wall modification in TEs is also completed with extreme changes in composition and thickness. In contrast, there is a lack of extensive cell wall elaborations in SEs; however, the width of cell wall increases and some components are inserted during SE differentiation as well (Mullendore *et al.*, 2010; Knoblauch *et al.*, 2016; Liesche *et al.*, 2017; Torode *et al.*, 2018).

Interspecific analyses of cell wall composition have reported weak and often contradictory findings regarding the evolutionary basis of cell wall formation and complexity. Starting with similarities, AGPs, as a large heterogeneous family of glycoproteins enriched in arabinose and galactose residues, were present in various root tissues, including vascular ones (Casero *et al.*, 1998; Marzec *et al.*, 2015; Marzec-Schmidt *et al.*, 2019). We confirmed this in roots of all studied species belonging to different evolutionary lineages. In all of them, the studied proteins were localized in phloem cells, which might suggest an important role of AGPs in phloem formation and functioning. However, no details are available about the role of AGPs in phloem cells other than structural. More often, the role of AGPs is considered in the context of xylem cell differentiation and secondary cell wall deposition, e.g. in the cell wall thickenings of future tracheids of vascular bundles (Schindler *et al.*, 1995; Altaner *et al.*, 2010). It has also been suggested that AGPs play a role in triggering cell death during the PCD process activated during xylem formation (Greenberg, 1996). Considering that in SEs, most cellular components are removed during phloemogenesis, and this process is referred to as semi-PCD (van Bel, 2003; Yang *et al.*, 2015), the localization of AGPs in phloem cells might also trigger the activation of degradation processes.

Another component, the β-1,4-galactans, are polysaccharides of plant cell walls, which, as we indicate, are a common and predominant component of the cell wall of phloem cells in various plant species, including ferns and angiosperms. The localization of β-1,4-galactan was determined in *A. thaliana* inflorescence stems and *Miscanthus giganteus* stems, including phloem SEs, and companion cells (Torode *et al.*, 2018). Phloem-specific localization of β-1,4-galactan was also confirmed in *C. richardii* roots and petioles (Eeckhout *et al.*, 2014). However, knowledge about the specific function of β-1,4-galactan in the primary cell wall remains limited. Several studies have shown that β-1,4-galactan plays an important role in modulating the mechanical properties of the cell wall (Ulvskov *et al.*, 2005). In *A. thaliana*, reduced galactan content resulted in thinner stems in comparison to the wild type (Obro *et al.*, 2009), with a noticeable impact on mechanical properties (McCartney *et al.*, 2000), that might also be confirmed by the widespread presence in phloem fibres and secondary xylem cells in the stem of *Populus trichocarpa* (Marzec-Schmidt *et al.*, 2019). Moreover, β-1,4-galactan is a water-retaining viscoelastic component of the cell wall that increases cell wall flexibility. This seems to be extremely important considering the function of the sieve tube, because a more flexible cell wall is better adapted to the tension induced by changes in turgor (Torode *et al.*, 2018; Yan *et al.*, 2021); likewise, xyloglucan, which increases tissue tension and determines changes in cell shape during differentiation (Hayashi and Kaida, 2011).

In our study, xyloglucan was present, especially in thickenings of TEs in roots of *C. richardii* and *A. thaliana*. However, so far, this component in *C. richardii* has been observed mainly in SEs (Eeckhout *et al.*, 2014), and a similar result was also reported in this work for *A. thaliana*. Xyloglucan was also present in the xylem of *Z. mays* roots and slightly in *Picea sitchensis*. There was a lack of this component in TEs of primary xylem in *Populus trichocarpa* roots; however, it is present in secondary structures (Marzec-Schmidt *et al.*, 2019). Xyloglucan found in xylem is responsible for generating tensile stress to bend plant organs. The function of xyloglucan in phloem cells is unknown; however, as for xylem, this cell wall compound was localized in phloem of wild-type *Populus alba* and was absent in phloem of transgenic plants overexpressing xyloglucanase (Baba *et al.*, 2009).

Arabinan contributes to the mechanical properties of the cell wall, providing flexibility and matrix porosity (Verhertbruggen *et al.*, 2009). The distribution of this compound in vascular tissues is diverse. A signal was detected only in the xylem and phloem of *Z. mays* roots and in immature xylem cells in the roots of *Populus trichocarpa*. However, in this tree species, small amounts of arabinan were also present in the phloem, which was confirmed, but at a different stage of root development (Marzec-Schmidt *et al.*, 2019).

Pectic HG is not only responsible for remodelling to facilitate cell wall mobility, binding capacity and structural changes, but also interacts dynamically with other polymers. Recently, HG has been recognized as a signalling molecule in cell wall integrity pathways, playing various roles in local changes in tissue softness during differentiation (Du *et al.*, 2022). Homogalacturonan incorporation and degradation in cell walls drive tissue or organ morphogenesis (Du *et al.*, 2022). This pectin determines cell wall properties, such as thickenings during wood formation (Siedlecka *et al.*, 2008; Biswal *et al.*, 2015) and at the contact site with microbial organisms (Leroux *et al.*, 2011). Interestingly, our studies revealed that this compound is always present in the primary structure of phloem in vascular plants. In roots, there was no binding of LM18 in xylem, but it was present in phloem in all analysed species, excluding *C. richardii*. In *Populus trichocarpa* roots, HG was observed in xylem only in the secondary structure (Marzec-Schmidt *et al.*, 2019). Likewise, JIM5 labelling of HG with a low degree of methyl esterification was observed in TEs of *A. thaliana* stem in the primary structure (Orfila *et al.*, 2005).

In summary, we confirmed the occurrence of AGPs and β-1,4-galactan in roots of all studied species belonging to different evolutionary lineages, suggesting their important role in phloem formation or function. In contrast, there was no similar pattern for other components, including xyloglucan, arabinan and HG, although AGP and HG are common components in other tissues. This suggests multifaceted roles mainly for polysaccharides, providing additional impetus to determine their function in the development and functioning of conducting elements. Intriguingly, their incorporation, removal and cooperation can completely change cell wall properties. Components of cell walls vary between tissues, species and even organs, in addition to their primary or secondary structures. It is worth noting that different antibodies can also detect different results for the distribution of cell wall components, and in some cases, HG can mask other epitopes, leading to problems with this kind of research (Marcus *et al.*, 2008). In these cases, the digestion of HGs as a control or evaluation of various antibodies can sometimes be necessary. The lack of specific patterns of cell wall modification during plant history suggests that evolution of this process is highly branching, with multiple successor mechanisms, in comparison to autophagy, which is clearly conserved. Considering dead TEs that transport water and minerals upwards or living SEs that allocate sugars downwards, it is an important area of inquiry to gain a better understanding of the chemical composition of their cell walls, which provide mechanical properties. Moreover, it suggests that the molecular framework of vascular tissue differentiation connects cell wall formation and PCD mechanisms.

Despite the importance of phloem, there is a lack of comprehensive literature data regarding the involvement and mechanism of organelle degradation pathways in the process of differentiating its conductive elements (SEs), in addition to the cell wall composition and its biosynthetic pathways. No detailed evolutionary analyses include phloem development, which seems necessary for understanding the evolution of components advancing transpiration and assimilation. Considering this, we have highlighted a strong involvement of autophagy in phloemogenesis. Conversely, we did not detect any effect of cell wall diversity selection on phloem functionality among species. In addition to these aforementioned scientific inquiries, our findings underscore the need for further investigation of multiple mutants with disrupted pathways in both autophagy and polysaccharide synthesis. Comprehensive studies examining these two processes would assist in identification of cooperative evolutionary drivers in the evolution of vascular tissues.

## SUPPLEMENTARY DATA

Supplementary data are available at *Annals of Botany* online and consist of the following.

Table S1: fragments of roots selected for analysis.

Table S2: sequences encoding ATG8 proteins in selected plant species.

Table S3: list of monoclonal antibodies for detection of cell wall components, with the epitopes that they recognize. All tested antibodies were characterized by high reactivity and specificity towards studied species. The pattern of signal distribution in the vascular cylinder is presented in Table 1.

Figure S1: a multiple alignment of the full-length amino acid sequences generated using ClustalW alignment in MEGA 7.0.

Figure S2: the unrooted neighbour-joining phylogenetic tree constructed in MEGA 7.0, with 500 bootstrap replicates, using 35 nucleotide sequences of ATG8 proteins from *Ceratopteris richardii* (starting as KAH), *Picea sitchensis* (EF), *Zea mays* (FJ), *Arabidopsis thaliana* (AT), and *Populus trichocarpa* (Potri). Different species are represented by different colours.

Figure S3: test of immunolocalization for arabinogalactan protein (AGP) and homogalacturonan (HG) using different antibodies in the pioneer roots of *Populus trichocarpa*. Red colour indicates lignin autofluorescence. Scale bars: 50 μm.

Figure S4: representative images of negative control reactions with omitted primary antibodies. Abbreviation: LM, light microscopy. Scale bars: 50 μm.

mcae015_suppl_Supplementary_Tables_S1

mcae015_suppl_Supplementary_Tables_S2

mcae015_suppl_Supplementary_Figures_S1

mcae015_suppl_Supplementary_Figures_S2

mcae015_suppl_Supplementary_Tables_S3

mcae015_suppl_Supplementary_Figures_S3

mcae015_suppl_Supplementary_Figures_S4

## Abbreviations

AG: arabinogalactan
AGP: arabinogalactan protein
BSA: bovine serum albumin
GFP: green fluorescent protein
HG: homogalacturonan
PBS: phosphate-buffered saline
PE: phosphatidylethanolamine
TSA: tyramide signal amplification
