# Supplementary material for: Conserved autophagy and diverse cell wall composition: unifying features of vascular tissues in evolutionarily distinct plants
Source: Ann Bot. 2024 Feb 7;133(4):559–72. doi: 10.1093/aob/mcae015 (PMC11037490; doi:10.1093/aob/mcae015)
Supplement: mcae015_suppl_Supplementary_Tables_S1 [file mcae015_suppl_supplementary_tables_s1.docx]

**Table S1.** Fragments of roots selected for analysis.

| **Species** | **Distances from the apex** |
| --- | --- |
| *Ceratopteris richardii* | 0.8 – 1.5 cm |
| *Picea sitchensis* | 2 – 3 cm |
| *Zea mays* | 1.5 – 2.5 cm |
| *Arabidopsis thaliana* | 1 – 2 cm |
| *Populus trichocarpa* | 1.5 – 3 cm |
