# Supplementary figures and images for: Conserved autophagy and diverse cell wall composition: unifying features of vascular tissues in evolutionarily distinct plants
Source: Ann Bot. 2024 Feb 7;133(4):559–72. doi: 10.1093/aob/mcae015 (PMC11037490; doi:10.1093/aob/mcae015)

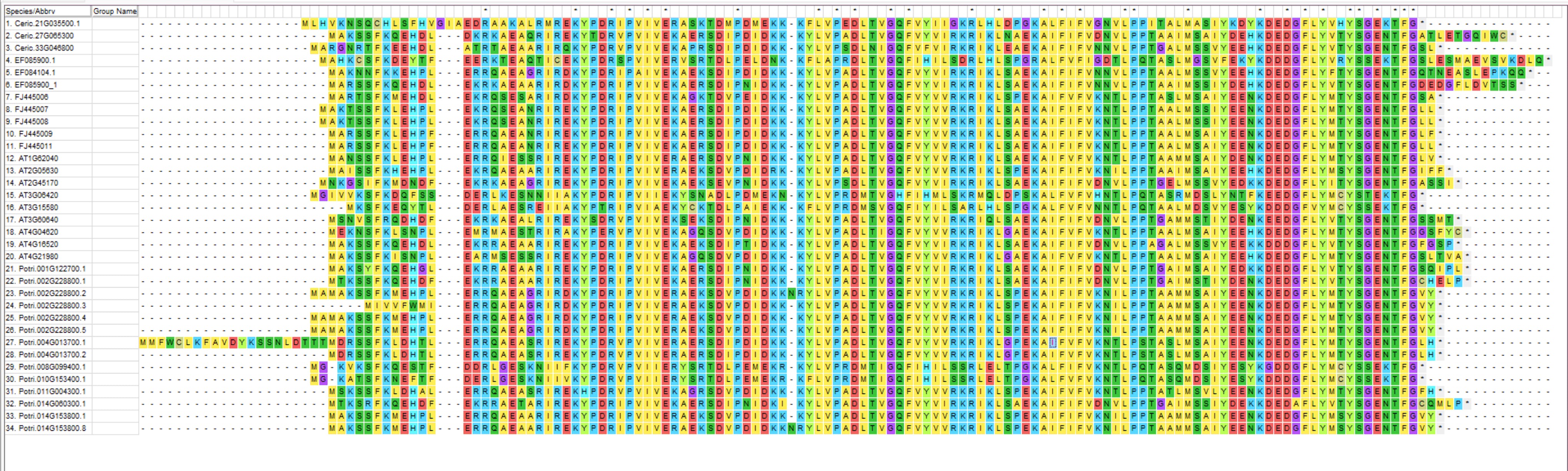

Supplement: mcae015_suppl_Supplementary_Figures_S1 [file mcae015_suppl_supplementary_figures_s1.jpeg]

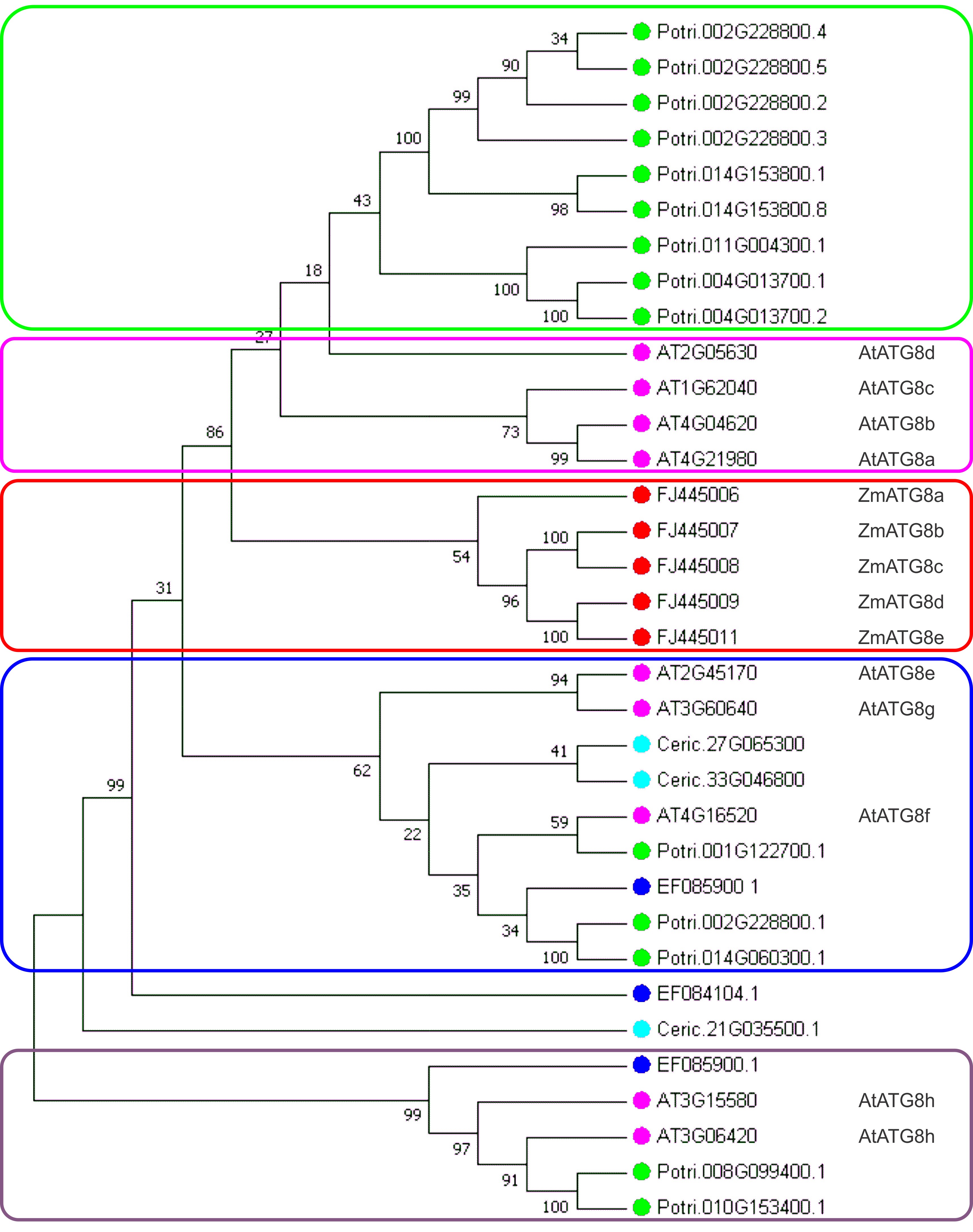

Supplement: mcae015_suppl_Supplementary_Figures_S2 [file mcae015_suppl_supplementary_figures_s2.jpeg]

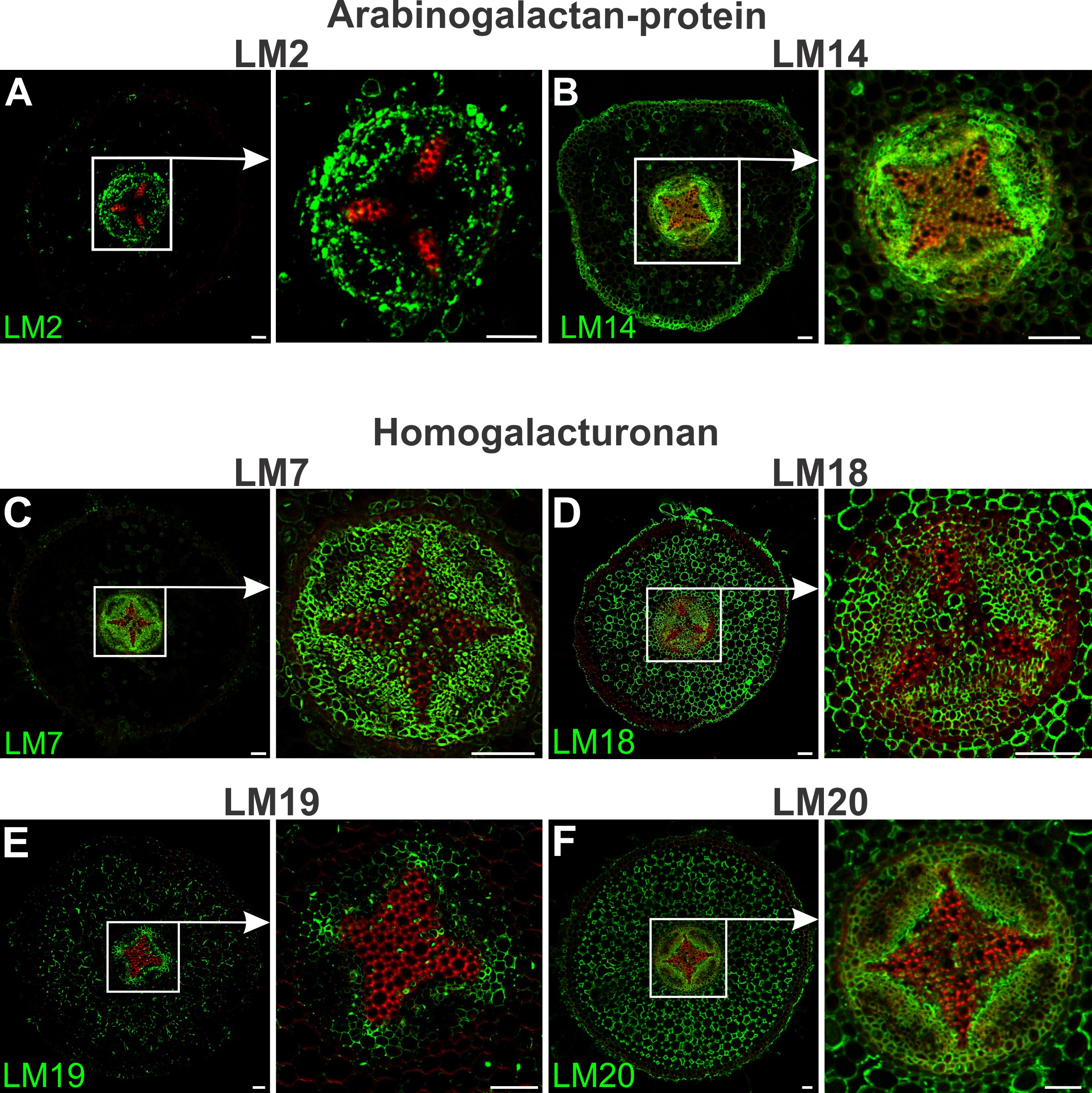

Supplement: mcae015_suppl_Supplementary_Figures_S3 [file mcae015_suppl_supplementary_figures_s3.jpeg]

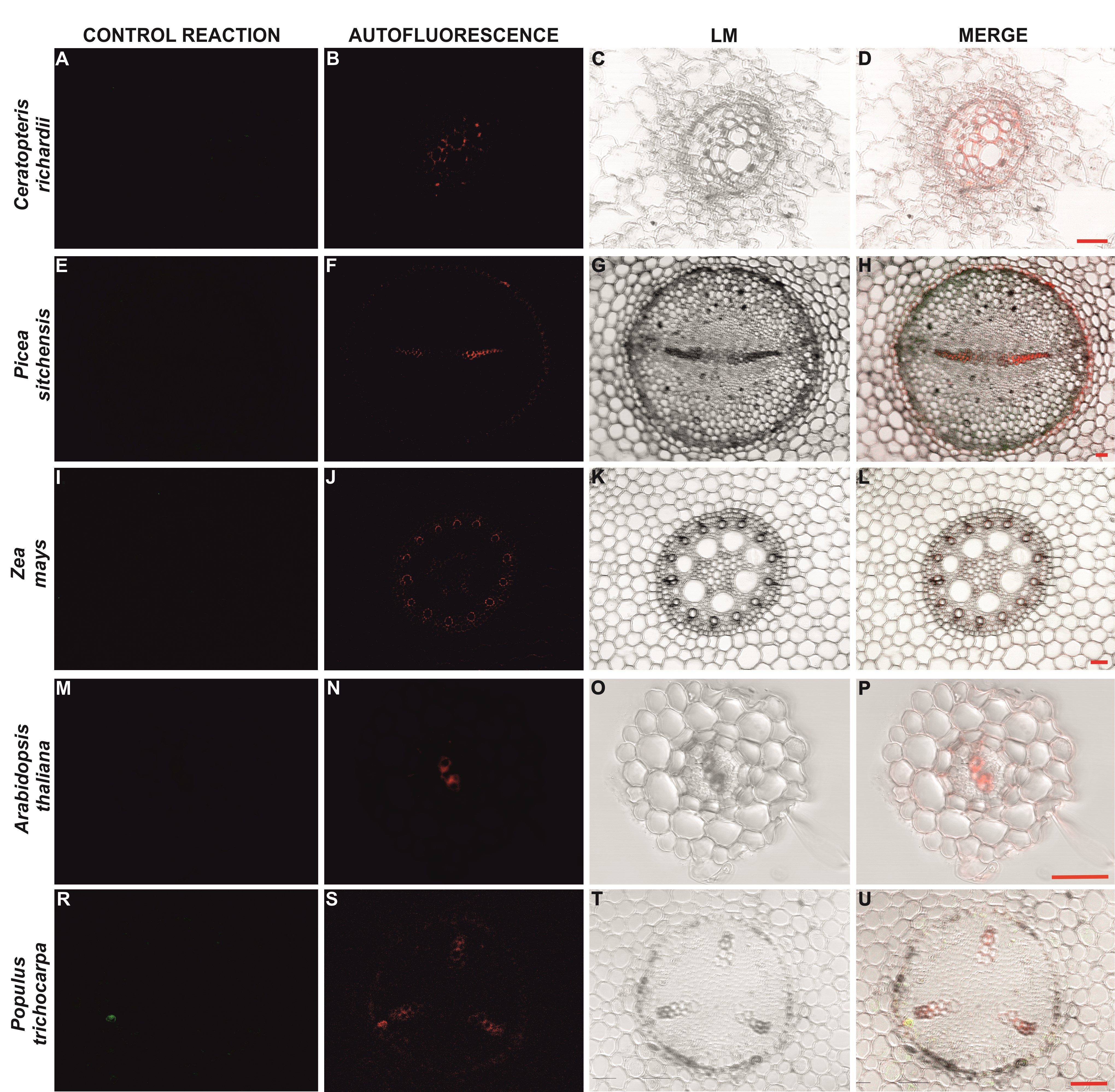

Supplement: mcae015_suppl_Supplementary_Figures_S4 [file mcae015_suppl_supplementary_figures_s4.jpeg]
