# Supplementary material for: Conserved autophagy and diverse cell wall composition: unifying features of vascular tissues in evolutionarily distinct plants
Source: Ann Bot. 2024 Feb 7;133(4):559–72. doi: 10.1093/aob/mcae015 (PMC11037490; doi:10.1093/aob/mcae015)
Supplement: mcae015_suppl_Supplementary_Tables_S3 [file mcae015_suppl_supplementary_tables_s3.docx]

**Table S3:** List of monoclonal antibodies for cell wall component detection with the epitopes that they recognize. All tested antibodies were characterised by high reactivity and specificity towards studied species. Pattern of signal distribution in vascular cylinder is presented in Table 1.

| **antibody ID** | **epitope** | **species tested** |
| --- | --- | --- |
| LM2 | β-linked-glucuronosyl residue of AGP | *Cr*, *Ps*, *Zm*, *At*, *Pt* |
| LM5 | (1-4)-β-D-galactan | *Cr*, *Ps*, *Zm*, *At*, *Pt* |
| LM7 | partially methyl-esterified homogalacturonan | *Pt* |
| LM14 | glucuronosyl residue of AGP | *Pt* |
| LM15 | xylosyl residues in the XXXG motif of xyloglucan | *Cr*, *Ps*, *Zm*, *At*, *Pt* |
| LM16 | galactosyl residues of (1-5)-α-Larabinan backbone | *Cr*, *Ps*, *Zm*, *At*, *Pt* |
| LM18 | partially methyl-esterified homogalacturonan | *Cr*, *Ps*, *Zm*, *At*, *Pt* |
| LM19 | unesterified homogalacturonan | *Pt* |
| LM20 | methyl-esterified homogalacturonan | *Pt* |
| *Cr* – *Ceratopteris richardii*, *Ps* – *Picea sitchensis*, *Zm* – *Zea mays*, *At* – *Arabidopsis thaliana*, *Pt* – *Populus trichocarpa* | | |
